# The current Monkeypox outbreak: Updates and concerns

**DOI:** 10.1016/j.jtumed.2022.08.001

**Published:** 2022-08-26

**Authors:** Abdurrahman Najeeb, Youssef M. Eltalkhawy, Omnia Reda

**Affiliations:** a Tropical Medicine Department, Faculty of Medicine, Alexandria University, Egypt; b Division of Infection and Hematopoiesis, Joint Research Center for Human Retrovirus Infection, Kumamoto University, Japan; c Department of Microbiology, Medical Research Institute, Alexandria University, Egypt; d Division of Genomics and Transcriptomics, Joint Research Center for Human Retrovirus Infection, Kumamoto University, Japan; e Department of Microbiology, High Institute of Public Health, Alexandria University, Egypt

**Keywords:** Africa, Control, Diagnosis, Monkeypox, Prevention, Transmission

## Abstract

Since 1958 witnessed the detection of Monkeypox virus in monkeys, no human infection was encountered until 1970. Afterwards, zoonotic transmission was the rule near African rainforests, mainly in DRC. Most cases occurred in children who weren’t immunized against smallpox. Since 2003 and the first human infection in the USA, research was accelerated. Two clades were identified with different virulence, demographic distribution and transmissibility. The mean age of infection increased with waning smallpox vaccine immunity. Mild febrile prodrome can precede lymphadenopathy, which doesn’t occur in smallpox. Homogenous crops of lesions appear in stages until scabs fall and contagiosity ends. However, since May outbreak, cases started to appear in non-endemic areas, human transmission increased and was linked to close sexual contact especially in MSM community. Lesions were found mainly perioral, at genitals and perianal. Newer system for nomenclature was suggested in which there are 3 viral clades and the responsible clade for the outbreak is clade 3 (lineage B.1). About 50 mutations were detected compared with the strains isolated 4 years ago. Gene loss and APOBEC3 may be related to accelerated mutation rate which may accelerate human transmission. Previous mistakes in failure to allocate available vaccines to control the disease in previously endemic areas should be avoided and rapid ring vaccination of potential contacts and those at risk should be a priority. Case isolation, contact isolation or tracing for an incubation period, standard measures for airborne infections and safe sex should be implanted in the light of the current uncertainty.

## Background

In 1980, after the world health organisation (WHO) declared that smallpox was globally eradicated, the other member of the *Orthopoxvirus* genus that remained of concern was Monkeypox. Both are double stranded DNA viruses with similar clinical features.

In May 2022, while the world was still convalescent from a tough COVID-19 pandemic, an outbreak of Monkeypox virus (MPXV) blurred and confused many health care personnels. Monkeypox is known about half a century ago, so what's new here?

Unlike smallpox, which is a strictly human disease, Monkeypox is a zoonotic disease. The discovery of the virus took place in research cynomolgus monkeys in Denmark that were imported from Singapore in 1958. The first human infection was confirmed in an infant in 1970. Afterwards, the virus was known to be endemic in western and central Africa, mainly in the former Zaire (the DRC) and surrounding countries. It was observed in remote villages near the tropical rainforests humans come into close direct contact with animals as squirrels, rodents, monkeys and apes or their body fluids. Back then, the high coverage of smallpox vaccination programs provided cross-immunity against Monkeypox virus. As a result, children, who were not vaccinated, were the main age group identified.^1^

## Spread outside Africa

The year 2003 witnessed the first human case outside Africa in the USA and was attributed to imported infected dogs (that were housed with Gambian rats). Afterwards, there was a shift in epidemiological trends in Africa with increasing mean age and waning of the cross-immunity in the community. Of note, MPXV has two clades with slight genomic difference: Central African Congo Basin (CB) clade of the virus appeared to be easily transmissible and more virulent compared to the West African (WA) clade. New classification proposed 3 clades; 1(CB), 2 & 3 (WA).^2^

## Clinical presentation

Classically, after 1–2 weeks of incubation, the clinical presentation begins by a 1–2-day febrile prodrome with headache and body aches. Lymphadenopathy, which may be cervical, femoral or inguinal, characterizes Monkeypox (and differentiates it from smallpox). Homogenous crops of macules, papules, vesicles, pustules and later crusts appear mainly on face and extremities, with palms and soles affected. Once scabs fall, usually after 2–4 weeks, contagiosity ends. Dysphagia has been reported. In most cases, it's a self-limiting disease. Mortality rate was classically <10%, predominantly in unvaccinated severe cases.^3^

## Concerns to health system

**Figure 1:**
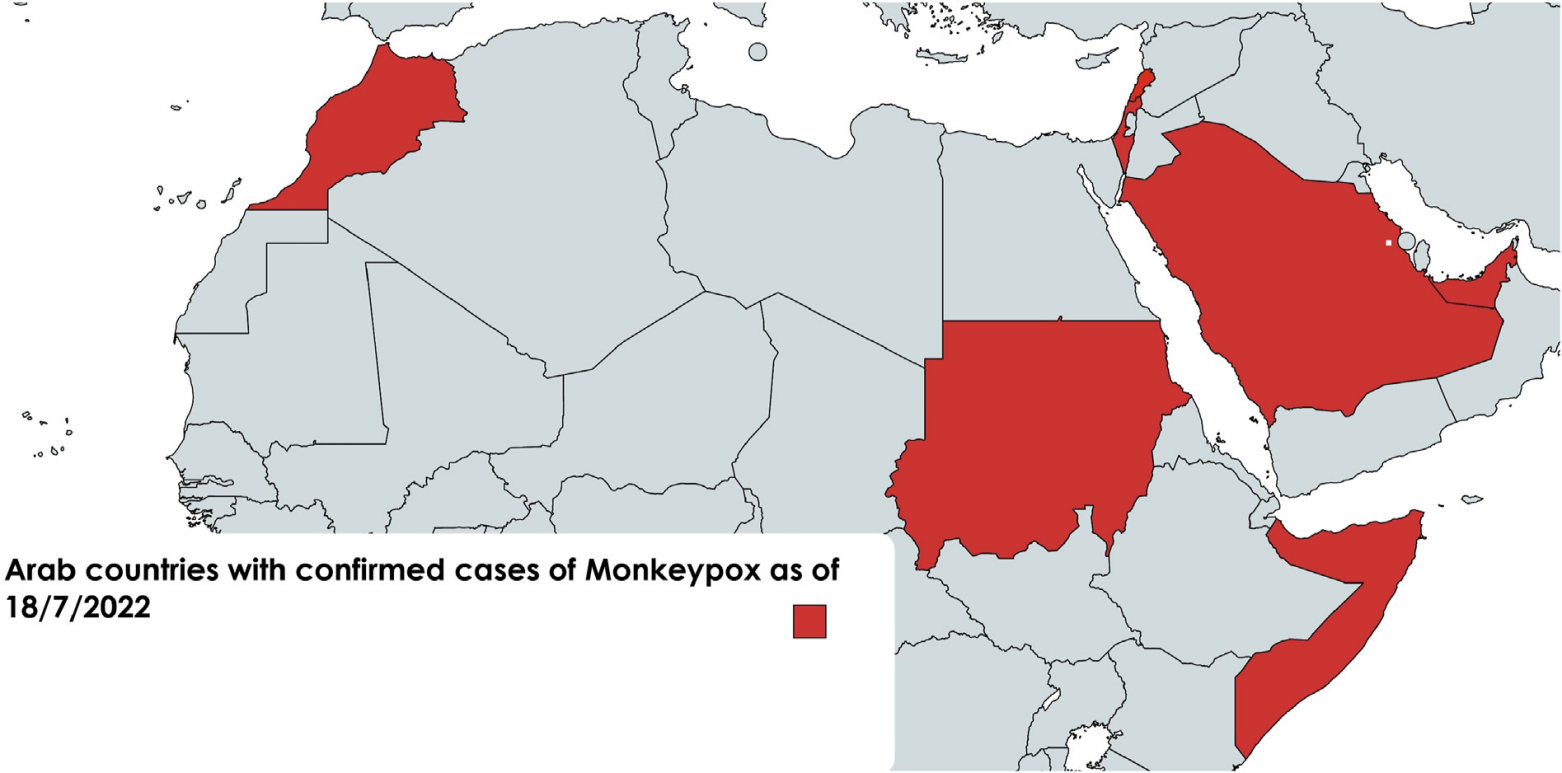
Arab countries where cases of Monkeypox virus infection were detected.

In the current outbreak started in May, there was a stupendous change in geographical distribution. Cases escalated in non-endemic areas including Europe, with high incidence in UK, Spain, Germany and Portugal, together with the USA. In the Arab world, cases were reported recently from UAE, KSA, Sudan, Lebanon, Morocco and Sudan (Figure 1). More than 12,500 cases were reported worldwide since May (www.monkeypoxmeter.com). Few cases reported travel to Africa. Earlier this year, there was a surge in the number of cases in endemic countries with 1238 cases reported in the DRC in the first 5 months of 2022.^4^

Another change noted in the current outbreak is the tendency of the virus to circulate in humans, with human-to-human transmission being the predominant mode of transmission, rather than the usual animal-to-human transmission. Many cases in the current outbreak in Europe and USA were associated with sexual encounters among “men who have sex with men” (MSM). Many other infections that used to spread by other routes, also showed recent sexually-transmitted outbreaks among sexual networks e.g., drug-resistant *Shigella* and hepatitis A, which highlights the importance of public health interventions and genomic surveillance.^5^

Many current cases also showed mild or absent prodrome. Moreover, the rash affects more genital, perianal and perioral areas rather than its classical distribution, suggesting that direct contact during intercourse is the expected method of transmission. It's noteworthy that reports from the last few years suggested possible sexual transmission.^6^

For diagnosis, samples include swabs/biopsies from skin/mucosal lesions and body fluids and blood samples. RT-PCR is the gold standard for definitive diagnosis. Sequencing allows for proper clade identification. Serological assays may help but they are not specific for MPXV as cross-reactivity with other orthopox viruses occur.^3^

The clade isolated in the current outbreak is the less pathogenic WA (clade 3- lineage B.1)^2^ with 1 reported death but the outbreak strains showed ∼50 new SNP mutations compared with the classical strains isolated 4 years ago.^7^ Gene loss mutations could be a factor behind the viral adaptation to human hosts and the spread of the current outbreak.^8^ Although APOBEC3 enzymes were suggested to account for this accelerated mutation rate of the DNA MPXV in the context of the current outbreak, its consequences and effect on human transmissibility or virus virulence remain to be elucidated.^2^

Antivirals available include IV Cidofovir, oral Brincidofovir (both act on DNA viruses by inhibiting their DNA polymerase) and Tecovirimat (it can be administered intravenously or as oral capsules; it inhibits orthopox viral exit from infected cells by targeting F13L gene of the p37 envelope protein), but their role is uncertain in Monkeypox and treatment is mainly supportive.^9^

Prevention is of utmost importance. Standard precautions to avoid direct contact with infected animals or humans should be maintained without ignoring the potential of airborne transmission. Patients are better kept in negative pressure rooms and contacts should be monitored/isolated for 21 days. Although it can survive for days in dry cold environment, it's susceptible to organic solvents (e.g. phenol and formaldehyde) and heat (20 min at 56 °C).^10^ Vaccine stocks have to be ready for any unexpected events. As the Vaccinia virus vaccine (2nd generation vaccine) rarely replicates in the recipient, live non-replicating MVA-BN (modified Vaccinia Ankara-Bavarian Nordic) strain vaccine (3rd generation vaccine) was developed. FDA approved MVA-BN as “Jynneos” vaccine in 2019 (Imvanex in EU).^11^ CDC currently recommends post-exposure vaccination and Vaccinia IVIG (for immunocompromised patients as live vaccines are contraindicated).^12^ Ring vaccination of all potential contacts is highly recommended.^13^

Many countries decided to offer vaccination to high-risk groups. However, lack of accurate surveillance system and failure to allocate available resources (including vaccines) in African countries earlier, raise concerns about health equity. Earlier measures to control MPXV in endemic areas could have been cost-effective and substantially decreased the possibility of occurrence of the current outbreak.

## Source of funding

This research did not receive any specific grant from funding agencies in the public, commercial or not-for-profit sectors.

## Conflict of interest

The authors have no conflict of interest to declare.

## Ethical approval

The study does not involve human subjects and/or animals.

## Authors contributions

**AN**: Conceptualization, Writing - Original Draft, review and edited the final draft. **YME:** Methodology, Data collection, Writing – review & editing. **OR:** Validation, Methodology, Writing – review & editing. All authors have critically reviewed and approved the final draft and are responsible for the content and similarity index of the manuscript.

## Data availability statement

Data sharing is not applicable to this article as no new data were created or analyzed in this study.
